# Dr. Horatio Gilbert

**Published:** 1915-06

**Authors:** 


**Dr. Horatio Gilbert,** Albany 1867, of Hornell (not listed in State Directory), died in Bath, April 26, aged 80. He served in the 157th N. Y. Volunteers.

